# The Potential of Glioblastoma Patient Symptoms to Diagnose and Predict Survival

**DOI:** 10.7759/cureus.16675

**Published:** 2021-07-27

**Authors:** Oliver D Mrowczynski, Ae L Yang, Jiangang Liao, Elias Rizk

**Affiliations:** 1 Neurosurgery, Penn State Health Milton S. Hershey Medical Center, Hershey, USA; 2 Public Health Sciences, Penn State Health Milton S. Hershey Medical Center, Hershey, USA; 3 Neurological Surgery, Penn State Health Milton S. Hershey Medical Center, Hershey, USA

**Keywords:** glioblastoma, glioma, symptoms, diagnosis, prognosis, survival

## Abstract

Glioblastoma is a devastating malignancy with a dismal survival rate and median survival time of 14 months. Currently, the biomarkers for glioblastoma are mostly molecular and include EGFRvIII, ATRX, PTEN, IDH1, MGMT, and others. These prognostic tumor biomarkers are obtained through a surgical biopsy and thus are not easily attainable. Clinicians would benefit from a robust, non-invasive, and readily available indicator for early diagnosis and accurate prognostication for glioblastoma patients. In this study, we assessed whether specific patient symptoms could provide an early diagnosis of glioblastoma. Further, we also assessed if any patient symptomatology could provide clinicians with the ability to prognosticate patient survival more accurately. We retrospectively reviewed the clinical data for 218 patients. We determined whether symptoms including headache, weakness, seizure, memory loss/confusion, visual changes, speech changes, and loss of consciousness led to a patient being diagnosed earlier and if any of these symptoms predicted diminished patient survival. Our study determined that weakness and memory loss/confusion were the symptoms that predicted diminished survival, and weakness alone was the symptom that predicted an earlier diagnosis. This study further elucidates the complexities of glioblastoma and provides clinicians with more data for their patients when discussing prognostication after diagnosis of glioblastoma.

## Introduction

Glioblastoma is a malignant brain tumor in adults with a dismal survival rate and median survival time of 14 months [1]. Glioblastoma, unfortunately, affects five per 100,000 people, making it the most common malignant brain tumor in adults [2]. Currently, the standard protocol for treatment includes maximal safe surgical resection with adjuvant chemotherapy of temozolomide (Temodar, MERCK & CO., Inc, Whitehouse Station, NJ) and radiation in large doses [3]. The current prognostic markers for glioblastoma are molecular and are obtained through a surgical biopsy. These biomarkers include EGFRvIII, ATRX, PTEN, IDH1, MGMT, and others [1,3-9]. Some are also currently used as markers to prognosticate patients and predict response to therapies [10,11]. Markers that are easily clinically attainable and can provide a robust prognostic indicator and early diagnosis would be beneficial for patients suffering from glioblastoma.

This study assesses whether symptoms including headache, weakness, seizure, memory loss/confusion, visual changes, speech changes, and loss of consciousness lead to a patient being diagnosed earlier and if any of these symptoms can predict diminished patient survival. Patient's clinical exam is easily attainable, thus making it an optimal marker. Understanding the relationship between glioblastoma patient symptomatology and diagnosis and prognosis may pave the way to utilizing symptoms as a robust and quick factor for patient prognostication. It also provides clinicians with more data to use when discussing with patients their prognosis and more accurately provide information about their disease.

In this study, we retrospectively analyze glioblastoma patients treated at our institution, comparing the time of diagnosis and patient survival depending on their specific symptomatology. Specifically, this study analyzes whether specific symptoms can predict early diagnosis or predict a decreased survival. We further aim to elucidate the complexities of this devastating disease. This study helps determine the underlying nuances of glioblastoma. It provides clinicians more data to speak to patients when discussing prognostication after a diagnosis of glioblastoma.

This article was previously published as a preprint at https://www.researchsquare.com/article/rs-200717/v1 as Clinical Study: Mrowczynski O, Yang AL, Liao J, Langan S, Rizk E. The Predictive and Diagnostic Potential of Symptoms for Glioblastoma Patient Survival. DOI: 10.21203/rs.3.rs-200717/v1.

## Materials and methods

Patient population

A retrospective chart analysis that included 218 histopathology-confirmed and diagnosed glioblastoma patients at the Pennsylvania State University Department of Neurosurgery from 2006 until 2016 was included in the study. The patients in this study were all pathologically confirmed to have WHO grade-IV glioblastoma multiforme. Most patients had the standard protocol for glioblastoma treatment, including maximal safe surgical resection, chemotherapy with temozolomide, and high-dose 60Gy radiation. Patients who had a previous low-grade glioma and any therapeutic intervention for a previous low-grade glioma were excluded from the study. Patients whose date of death could not be accurately determined were excluded.

Data collection and statistical analysis

The dataset consisted of 218 subjects. Days from the first symptom to death, days from the first symptom to diagnosis, and days from the diagnosis to death were calculated for each subject. The dependence of these three time-to-event variables on various predictors was modeled using Cox's proportional hazards models. These predictors included age; sex; symptoms including headache, weakness, seizure, memory loss and confusion, visual changes, speech changes, loss of consciousness; and treatments, including resection, radiation, and Temodar. Additionally, days from the first symptom to diagnosis is also used as a potential predictor for days from diagnosis to death. Specifically, this study analyzes whether specific symptoms can predict an early diagnosis or decreased survival. All data in this study were subjected to statistical analysis. Our final Cox model for each time-to-event variable consists of only statistically significant predictors. The analysis was conducted using R 3.4 (R Foundation for Statistical Computing). A p-value < 0.05 was deemed statistically significant. This study received Penn State Health Milton S. Hershey Medical Center Institutional Review Board approval, IRB #5691.

## Results

Patient characteristics

The characteristics of our 218 patients are shown in Table 1.

**Table 1:** Patient Characteristics (N=218)

	N(%)
Median age, years (range)	64(5-88)
Sex:	
Male	110 (51)
Female	108 (49)
Symptoms:	
Asymptomatic	4 (1.8)
Headache	65 (29.8)
Weakness	77 (35.3)
Seizure	21 (9.6)
Memory loss/Confusion	86 (39.5)
Visual Changes	15 (6.9)
Speech Changes	55 (25.2)
Loss of Consciousness	4 (1.8)
Treatments:	
Resection	135 (61.9)
Radiation	148 (67.9)
Temodar	152 (69.7)

Patients' median age was 64 years old with a range of 5 to 88 years old. In our cohort, 110 patients were male, comprising 51% of our sample population, while 108 patients were female, comprising 49% of our sample population. We also described the number of patients with specific symptoms; some patients had multiple presenting symptoms. Four patients were asymptomatic, comprising 1.8% of our sample population. Sixty-five patients had a headache, comprising 29.8% of our sample population. Seventy-seven presented with weakness, comprising 35.3% of our sample population. Twenty-one had a seizure, comprising 9.6% of the population. Eighty-six had memory loss/confusion, comprising 39.5% of our sample population. Fifteen patients had visual changes, comprising 6.9% of our sample population. Fifty-five had speech changes, comprising 25.2% of our sample population. Four had a loss of consciousness, comprising 1.8% of our sample population. We also analyzed the data of the patients in our sample who were treated. One hundred and thirty-five patients had a surgical resection, comprising 61.9% of our sample population. One hundred and forty-eight received radiation, comprising 67.9% of our sample population, and 152 took Temodar, comprising 69.7% of our sample population.

Symptom prediction of survival

Patient symptoms as a predictor of survival is shown in Table 2.

**Table 2:** Predictor of Survival

	P Value	Hazard Ratio and 95% Confidence Interval
Age	3.17 x10^-5	1.027 [1.014-1.039]
Sex (male)	0.253	1.191 [0.883-1.607]
Symptoms:		
Headache	0.239	0.821 [0.592-1.14]
Weakness	0.0063	1.543 [1.131-2.106]
Seizure	0.206	0.717 [0.427-1.201]
Memory loss/Confusion	0.0588	1.345 [0.989-1.83]
Visual Changes	0.21	0.651 [0.332-1.274]
Speech Changes	0.642	1.084 [0.771-1.524]
Loss of Consciousness	0.626	0.707 [0.175-2.855]
Treatments:		
Resection	0.00085	0.593 [0.436-0.806]
Radiation	1.06 x10^-10	0.338 [0.243-0.47]
Temodar	<2 x 10^-16	0.222 [0.157-0.314]

**Figure 1:**
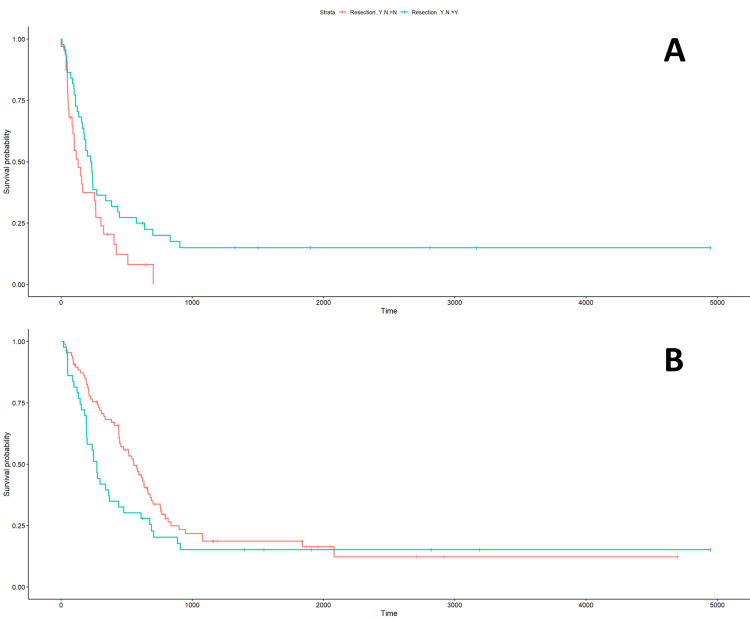
Effects of Weakness and Resection on Survival (1A) In the group of patients who had a weakness, not having a resection led to a decreased survival (P=0.04). (1B) In the group of patients who did have a resection, patients with weakness trended toward a shorter and decreased survival (P=0.08).

The older the patient, the greater the decrease in survival (P=3.17 x 10^-5, hazard ratio [HR] 1.027 [1.014-1.039]). Patients presenting with weakness had a decreased survival, which was statistically significant (P=0.0063; HR 1.543 [1.131-2.106]). Patients presenting with memory loss/confusion had a decreased survival, which trended toward statistical significance (P=0.0588; HR 1.345 [0.989-1.83]). All other symptoms, including headache, seizure, visual changes, speech changes, and loss of consciousness, did not predict survival. As a control for our study, we evaluated the predictor of survival when patients received treatment with resection, radiation, and/or Temodar. Patients who had a resection had an increased survival (P=0.00085; HR 0.593 [0.436-0.806]). Patients who had radiation and Temodar also had an increased survival (P=1.06 x 10^-10; HR 0.338 [0.243-0.47] and P=<2 x 10^-16; HR 0.222 [0.157-0.314], respectively). We also looked at the effects of resection and weakness. Figure 1A shows that not having a resection led to a lower survival probability (P=0.04). Figure 1B shows that patients with weakness in the group of patients who did have a resection tended toward a shorter and decreased survival probability (P=0.08).

Symptom prediction of diagnosis

Patient symptoms as a predictor of early diagnosis are shown in Table 3.

**Table 3:** Predictor of Diagnosis

	P Value	Hazard Ratio and 95% Confidence Interval
Age	0.625	0.998 [0.988-1.008[
Sex (male)	0.235	1.18 [0.898-1.551]
Symptoms:		
Headache	0.62	1.077 [0.804-1.444]
Weakness	0.0152	1.428 [1.071-1.904]
Seizure	0.891	1.0326 [0.657-1.622]
Memory loss/Confusion	0.344	0.875 [0.664-1.154]
Visual Changes	0.72	0.905 [0.526-1.559]
Speech Changes	0.636	0.927 [0.679-1.266]
Loss of Consciousness	0.151	0.483 [0.1784-1.305]

Age and sex were not found to be predictors of an earlier diagnosis. Patients who presented with weakness as a symptom had a statistically significant increase in their speed of diagnosis (P=0.0152; HR 1.428 [1.071-1.904]). All other symptoms, including headache, seizure, memory loss/confusion, visual changes, speech changes, and loss of consciousness, did not predict an earlier diagnosis.

## Discussion

Glioblastoma patients have a devastating prognosis, and non-invasive patient prognosis markers are important to help patients understand their disease and for the physician to more accurately predict a patient's survival. The patient’s symptoms are one of the most easily attainable factors of a glioblastoma patient presentation. The possibility of using these data to prognosticate better a patient's clinical course is something to be desired. The better characterization of a glioblastoma patient regarding symptomatology also helps to understand this complex disease further. These data help the clinician be more accurate and personalized when discussing a patient's prognosis and disease course. To our knowledge, this is the first study analyzing and characterizing symptoms with regard to glioblastoma patient diagnosis and prognosis.

Our data included controls, e.g., the patient's age and treatment with surgical resection and adjuvant chemoradiation. As is known, older patients have diminished survival, as shown in our data,. Additionally, surgical resection and treatment with adjuvant chemotherapy and radiation are known to increase survival, which is also shown in our data. These factors were used to demonstrate our patient cohort following the known trends in the literature.

As far as weakness is concerned as a symptom that predicted earlier diagnosis, this may be because it is easily recognizable by the patient or their family. A patient's family that lives with them is able to realize that something is wrong with their family member and brings them to the emergency department to be evaluated. Indeed, multiple other studies in different cancer types including prostate, breast, multiple myeloma, elderly glioma, colorectal, and head and neck cancers have looked at weakness and frailty as a predictor of patient survival [12-18]. These studies show that weakness is a predictor of poor outcome in cancer. Furthermore, studies have shown that cognitive deterioration precedes MRI progression [19]. Symptoms like visual changes may not bring the patient to be evaluated sooner as the patient may be in denial of the occurring changes. These findings are different than the Karnofsky Performance Scale (KPS) as KPS is a functional impairment scale and not specific to weakness as a presentation. As far as weakness and memory loss/confusion being a predictor for decreased survival is concerned, this may be because these symptoms cause significant decrease in daily functions. Previous studies have also looked at the test of verbal memory in glioma patients and found that verbal memory was independently and strongly associated with survival [20]. The patient being unable to care for themselves in the same way, which as before may lead to decreased patient survival overall. We show that in the group of patients who did have a resection, weakness was an independent predictor of poor outcome.

This study's limitations include its retrospective nature and that it only assesses the outcomes of patients in one institution. It also did not take into account a patient's financial status, which may have an impact on the availability of treatments and, thus, patient survival.

## Conclusions

This study demonstrates that weakness and memory loss/confusion were the symptoms that predicted diminished survival, and weakness alone was the symptom that predicted an earlier diagnosis. This study further helps to elucidate the complexities of glioblastoma and provides clinicians with more data which they can provide to patients when discussing prognostication after a diagnosis of glioblastoma. Further studies must be performed in larger cohorts in other institutions to confirm this finding further.
